# Use of mebendazole (anthelmintic) for recurrent uncomplicated urinary tract infections in a healthy female: A case report

**DOI:** 10.5339/qmj.2025.92

**Published:** 2025-09-22

**Authors:** Ibtisam Musameh, Zeinab Abdel Mohsen, Asmaa Mohamed

**Affiliations:** 1 Pharmacy Department, Hamad General Hospital, Hamad Medical Corporation, Doha, Qatar; 2 Hamad Medical Corporation, Doha, Qatar; 3 Hamad General Hospital, Hamad Medical Corporation, Doha, Qatar *Email: imusameh@hamad.qa

**Keywords:** Recurrent urinary tract infection, pinworm, anthelmintic, case report, mebendazole

## Abstract

**Background:**

Recurrent uncomplicated urinary tract infections (UTIs) in healthy females pose a significant challenge to both patients and healthcare professionals, due to the need for repeated antibiotic treatments and the recurrence of symptoms and suffering. This case report suggests a potentially simple approach to treating recurrent uncomplicated UTI in a healthy, sexually active female, which, if validated, may eliminate the need for frequent antibiotic courses and long-term prophylactic use, potentially leading to a complete cure.

**Case presentation:**

A 35-year-old healthy, sexually active Middle Eastern female was diagnosed with a recurrent uncomplicated UTI. The patient received multiple courses of culture-guided antibiotics, including ertapenem, for over a period of two years. The patient inadvertently recovered from the disease after she and her family received mebendazole treatment, following the diagnosis of a pinworm infestation in her five-year-old child. All patients’ symptoms resolved, with no recurrence of urinary tract infections or need for antibiotics during the two-year follow-up period after mebendazole administration. The patient remained sexually active throughout the follow-up period without any lifestyle modifications.

**Discussion:**

Recurrent urinary tract infections (rUTIs) in healthy females remain a significant medical challenge as there is no established hypothesis to explain why some women are more susceptible to rUTIs than others. While pinworm (*Enterobius vermicularis*) is a well-recognized parasite, research has primarily focused on its effect on children – with few studies investigating its impact on the adult population. This may be attributed to the fact that pinworm infections in adults are mostly self-limiting and asymptomatic. However, some case reports have documented complications arising from pinworm.

**Conclusion:**

This case raises the question of whether empirical treatment with mebendazole should be considered for recurrent uncomplicated UTIs in sexually active, healthy females – alongside standard antibiotic therapy. Identifying pinworm infection in healthy adult females with rUTIs could provide valuable insights, particularly if an association between the two is confirmed.

## 1. INTRODUCTION

Recurrent urinary tract infections (rUTIs) are defined as two or more infections within six months, or three or more within a year. rUTIs often present as acute simple cystitis. Women appear to be more susceptible to both simple cystitis and its recurrence due to a combination of host behavioral, anatomical, physiological, and genetic factors; however, none of these has been definitely proven to cause rUTI.^1–3^ The question of why some women are more likely, than others, to develop and sustain rUTI remains unanswered.^4^

Urinary tract infection (UTI) is a major concern for both patients and health system due to its high treatment costs and the emergence of multidrug-resistant bacteria. Each year, around 11 million cases of UTI are reported in the United States alone, resulting in approximately $5 billion in direct and indirect expenditures with an increasing overall cost over time.^5–8^ The cost of an acute antibiotic ranged from $10 for oral trimethoprim-sulfamethoxazole to $3,970 for intravenous ertapenem, which represents a great health burden.^9^

This case report presents a healthy 35-year-old, sexually active, monogamous woman who was diagnosed with recurrent uncomplicated UTIs. Informed consent was obtained from the patient, and the ethical approval for publication was obtained from the Hamad Medical Corporation Medical Research Center (MRC-04-24-619).

## 2. CASE PRESENTATION

A 35-year-old Middle Eastern woman with no significant past medical history presented to the urology clinic for evaluation. She is sexually active and has a history of four full-term pregnancies and deliveries (Gravidity 4, Parity 4). Two months prior to her visit, she underwent her second cesarean section. Since then, she has been experiencing rUTIs, confirmed by positive urine cultures. Her symptoms – including dysuria, urinary urgency, frequency, and a burning sensation during urination – have persisted despite completing multiple courses of antibiotics.

Ultrasound imaging revealed bladder wall thickening measuring approximately 0.7 cm, consistent with a history of persistent cystitis. Non-enhanced CT scans of the abdomen and pelvis showed no evidence of urolithiasis.

Over a two-year period following her initial presentation at the urology clinic, the patient was treated for recurrent uncomplicated UTIs with multiple culture-guided oral and intravenous antibiotics, including a carbapenem. Despite being well-educated and compliant with prescribed prophylactic measures – such as nitrofurantoin, which she discontinued after two months, and cranberry supplements – her symptoms persisted. She reported voiding every 2–3 hours and occasionally self-initiated short courses of antibiotics when symptoms worsened. Throughout this period, she never felt fully recovered and continued to experience varying degrees of UTI symptoms.

A significant turning point occurred when her five-year-old child was diagnosed with a pinworm infestation. The entire family, including the patient, was prescribed mebendazole. Following the administration of mebendazole, she reported complete resolution of her UTI symptoms, including burning sensation and foul-smelling urine. Remarkably, she remained symptom-free thereafter, without the need for further antibiotics or cranberry supplements.

Upon further inquiry, the patient revealed that her child had been experiencing intermittent anal itching for the past two years, coinciding with the onset of her own UTI symptoms. However, she had not sought medical attention for her child until recently. The patient also admitted experiencing occasional anal itching during the same period but had not reported it to her healthcare provider.

The patient and her family were each administered a 100 mg oral dose of mebendazole, which was repeated after two weeks. Following this treatment, the patient remained free of UTI symptoms and anal pruritus throughout a two-year follow-up period without any prophylactic measures.

## 3. DISCUSSION

Simple recurring UTIs remain a significant concern due to their associated costs, the need for regular medical follow-up, patient discomfort, and the potential for antibiotic resistance and multidrug-resistant bacteria.^1,6–8^

In the United States, 27% of women with uncomplicated cystitis experience a proven recurrence. Within six months of their initial UTI episode, 2.7% experience a second recurrence during the same period.^10^ No clear mechanism has yet been identified to explain the cause of UTI recurrence, despite multiple proposed theories.

It is suggested that there are host-associated reservoirs – such as the gastrointestinal tract and the underlying bladder tissue – that can contribute to rUTIs, as these infections are often caused by the same strain of *Escherichia coli* that induced the initial episode.^1^ Recent studies using advanced technologies are now focusing on the connections between the UTIs and the microorganisms in the gut, vagina, and urine.^11^ According to one of the most well-established explanations for rUTI, the gastrointestinal tract serves as a reservoir for uropathogens that are periodically reintroduced into the urinary tract through contamination of the periurethral area and subsequent retrograde ascent.^12^ In one study that compared the fecal and vaginal samples from UTI patients to healthy controls, 87% of patients had the UTI-causing bacterium in their fecal microbiota, indicating that the fecal reservoir plays a significant role in the pathogenesis.^13^

*Enterobius vermicularis* (pinworm) is a well-known common intestinal parasite that primarily infects the gastrointestinal system.^14^ The vulva, vagina, uterus, fallopian tubes, ovaries, and even the peritoneum via the fallopian tubes are uncommon sites of infestation. Other uncommon documented sites include the liver, breast, spleen, and the lung.^15–17^ In regions with a higher prevalence of enterobiasis, rUTIs have been associated with the transmigration of intestinal flora, including *Enterobius*, to the urine tract. We reviewed various case reports in the literature describing recurrent uncomplicated UTI associated with pinworm, which were successfully treated with anthelmintics, leading to resolution of the rUTIs.^18–20^ Some research indicates a link between pinworm and rUTI in young females,^21,22^ as well as an association between pinworm and enuresis in children.^23,24^ Although genitourinary complications are rare, they are a recognized risk associated with pinworm infections. However, no studies have been conducted to investigate the potential link between pinworm infestations and rUTIs in adult females. While our patient occasionally experienced anal pruritus, she never considered pinworm as the cause. She reported that her UTI symptoms most often worsened at night and following sexual activity.

Diagnosis of pinworm (*E. vermicularis)* is difficult because most adult patients are asymptomatic. Detection of the pinworm requires multiple tests. The scotch tape test, when performed only once, has a sensitivity of about 50%, but repeating it on three different days can increase the sensitivity to 90% – indicating that some patients may receive false-negative results.^25,26^

When a child is infected, household contacts are also at high risk of infection. Therefore, it is recommended to treat all the family members, even without testing. Adult patients are also vulnerable to infection, yet there is a lack of data on how they are really affected.

Transmission of bacteria from the rectum to the urinary tract via pinworm movement is one of the proposed hypotheses for rUTIs. Although the exact incidence of pinworm reinfection is unclear, it is possible because pinworm eggs have a comparatively high level of environmental resilience. They can survive on clothing, bedding, and other surfaces for 2–3 weeks.^27^ Reinfection can prolong the disease course, effectively making the rectum a reservoir for bacteria. In this case, the patient reported that her child experienced intermittent anal itching for two years without medical intervention. During this same period, the mother developed rUTIs which persisted until the entire family was treated with mebendazole.

A rUTI questionnaire may serve as a useful tool for assessing pinworm infection risk in healthy adult females. This questionnaire could include key indicators such as perianal pruritus, the presence of children in the household with anal itching, and other relevant factors. By identifying females at risk of pinworm infestation, healthcare providers can more accurately assess the need for mebendazole treatment and its potential impact on recurrent uncomplicated UTIs. If an association between pinworm infection and rUTI is confirmed, it could significantly impact the treatment approach for recurrent uncomplicated UTIs in sexually active females. Further research is necessary to validate the effectiveness of this strategy and its potential to improve clinical outcomes.

## 4. CONCLUSION

This case highlights a potential relationship between pinworm infection and recurrent uncomplicated UTIs in a sexually active, healthy adult female – an association that is not well studied in adults. The patient reported recovery from rUTIs after receiving mebendazole for pinworm eradication. Further studies are needed to determine whether the patient’s recovery was coincidental or a result of mebendazole administration. If mebendazole is proven effective, it could significantly impact the treatment of recurrent uncomplicated UTIs in healthy females.

## LIST OF ABBREVIATIONS

**Table:** 

UTI	Urinary Tract Infection
rUTIs	Recurrent Urinary Tract Infections
